# Tuberculosis infection prevention and control: why we need a whole systems approach

**DOI:** 10.1186/s40249-020-00667-6

**Published:** 2020-05-25

**Authors:** Karina Kielmann, Aaron S. Karat, Gimenne Zwama, Christopher Colvin, Alison Swartz, Anna S. Voce, Tom A. Yates, Hayley MacGregor, Nicky McCreesh, Idriss Kallon, Anna Vassall, Indira Govender, Janet Seeley, Alison D. Grant

**Affiliations:** 1 The Institute for Global Health and Development, Queen Margaret University, Edinburgh, EH21 6UU UK; 2 TB Centre, London School of Hygiene & Tropical Medicine, London, UK; 3 Division of Social and Behavioural Sciences, Faculty of Health Sciences, University of Cape Town, Cape Town, South Africa; 4 Discipline Public Health Medicine, School of Nursing and Public Health, University of KwaZulu-Natal, Durban, South Africa; 5 Department of Infectious Disease, Faculty of Medicine, Imperial College London, London, UK; 6 The Institute of Development Studies, University of Sussex, Brighton, UK; 7 Department of Global Health and Development, Faculty of Public Health and Policy, London School of Hygiene & Tropical Medicine, London, UK; 8 Africa Health Research Institute, School of Nursing and Public Health, University of KwaZulu-Natal, Durban, South Africa; 9 School of Public Health, Faculty of Health Sciences, University of the Witwatersrand, Johannesburg, South Africa

**Keywords:** Drug-resistant tuberculosis, Infection prevention and control, Health system, South Africa

## Abstract

Infection prevention and control (IPC) measures to reduce transmission of drug-resistant and drug-sensitive tuberculosis (TB) in health facilities are well described but poorly implemented. The implementation of TB IPC has been assessed primarily through quantitative and structured approaches that treat administrative, environmental, and personal protective measures as discrete entities. We present an on-going project entitled *Umoya omuhle* (“good air”), conducted in two provinces of South Africa, that adopts an interdisciplinary, ‘whole systems’ approach to problem analysis and intervention development for reducing nosocomial transmission of *Mycobacterium tuberculosis* (*Mtb*) through improved IPC. We suggest that TB IPC represents a complex intervention that is delivered within a dynamic context shaped by policy guidelines, health facility space, infrastructure, organisation of care, and management culture. Methods drawn from epidemiology, anthropology, and health policy and systems research enable rich contextual analysis of how nosocomial *Mtb* transmission occurs, as well as opportunities to address the problem holistically. A ‘whole systems’ approach can identify leverage points within the health facility infrastructure and organisation of care that can inform the design of interventions to reduce the risk of nosocomial *Mtb* transmission.

## Background

Two recent opinion pieces in leading public health journals underline the importance of holistic, multisectoral, and person-centred approaches to address challenges raised by antimicrobial resistance (AMR) [1, 2]. However, there are few examples of how this approach might translate in practice. To advance the agenda, we share our experience of applying a whole systems approach to an on-going study of infection prevention and control (IPC) for both drug-sensitive (DS-TB) and drug-resistant tuberculosis (DR-TB) in South Africa.

TB remains one of the most critical issues facing global public health and health systems today: the disease is responsible for over one million deaths every year, with DR-TB accounting for 29% of AMR-related deaths [3]. Health facilities are neglected sites of *Mycobacterium tuberculosis* (*Mtb;* considered here to include DS- and DR-*Mtb*, since they are indistinguishable prior to diagnosis) transmission [4], due to the convergence of people with TB and people with increased susceptibility to developing TB. Despite clear guidelines for TB infection prevention and control (IPC) that are equally relevant to DS-TB and DR-TB, there is only weak evidence to show that implementing TB IPC reduces nosocomial transmission. Though commonly referring to infection originating within hospitals, we apply the term more broadly to designate infection occurring within health facilities [5]. IPC measures to reduce airborne transmission of *Mtb* in health facilities, such as opening doors and windows, wearing protective respirators, and instituting cough triage, remain poorly implemented [6–8]; there are large gaps in understanding the barriers and enablers to implementing these measures in resource-constrained health systems and specifically, within primary health clinics.

## Main text

### Limited understanding of why TB IPC measures are poorly implemented

Recommendations to improve health care worker (HCW) adherence to guidelines tend to focus on training and supportive resources, yet there is limited understanding of which elements work to enable sustained implementation. Little attention has been paid to the complex contextual features of clinics (and of the wider health system) that underpin HCW understanding and implementation of TB IPC measures. This includes, for example, national and provincial policies governing the delivery of TB services (are they centralised or decentralised? integrated or stand-alone?); the architectural design and routine maintenance of health facilities (are infection control measures included?); occupational health and safety (are measures in place to protect HCWs from and compensate them for work-related infections?), and cross-cutting quality improvement efforts (is IPC part of routine audits and accreditation processes?). Equally critical are facility-based protocols and processes that shape the organisation of care, which affects *how long* and *where* patients spend time in clinics, in turn affecting the risk of nosocomial transmission.

These gaps in understanding the health systems context relevant to TB IPC are evident in South Africa: nosocomial transmission featured prominently in the 2005 outbreak of extensively drug-resistant (XDR) TB in a hospital HIV outpatient service in KwaZulu-Natal and is likely to remain a key driver of *Mtb* transmission in the country [9, 10]. South African TB IPC guidelines are in place [11], yet numerous studies report inadequate implementation in health facilities [8, 12]. Studies tend to adopt cross-sectional designs and survey methods to assess HCW knowledge, attitudes, and practices, reflecting the focus on ‘failed’ IPC as poor adherence to guidelines rather than symptomatic of root systemic issues. There is limited recognition of how national policies, as well as local working environments within which *Mtb* transmission is more or less likely to occur, impact on HCW and managerial practices in the implementation of TB IPC.

### Going beyond a narrow conception of implementation of TB IPC measures

Rather than viewing TB IPC implementation as largely driven by individual agency and discrete tasks, we suggest that TB IPC represents a ‘complex intervention’ with multiple, interacting components that require tailoring to specific settings [13], for example, clinic design, size, leadership culture, and patient load. TB IPC measures such as opening windows or wearing respirators are not only learned behaviours, but rather practices that have to become ‘routine’ within the day-to-day working environment and culture of primary care clinics. As the authors of a systematic review of HCW behaviour change interventions for IPC suggest, research in this area should ‘*understand the people practising the behaviour*’ and ‘*understand the setting in which people are practising the behaviour*’ [14].

Understanding both human behaviour and the organisation of care within the TB IPC practice environment requires an approach that focuses on the clinic as a dynamic site of interaction between humans, microbes, and materials. On the one hand, clinics are microcosms, i.e., bounded areas that are governed by delineated spaces and timing, as well as codes of conduct. On the other hand, they are permeable units through which people and their ideas about risk, infection, and transmission, flow. Further, local clinic dynamics are influenced by broader changes in the health systems, funding, and policy environments.

### *Umoya omuhle*: a whole systems approach to TB IPC in South Africa

**Fig. 1:**
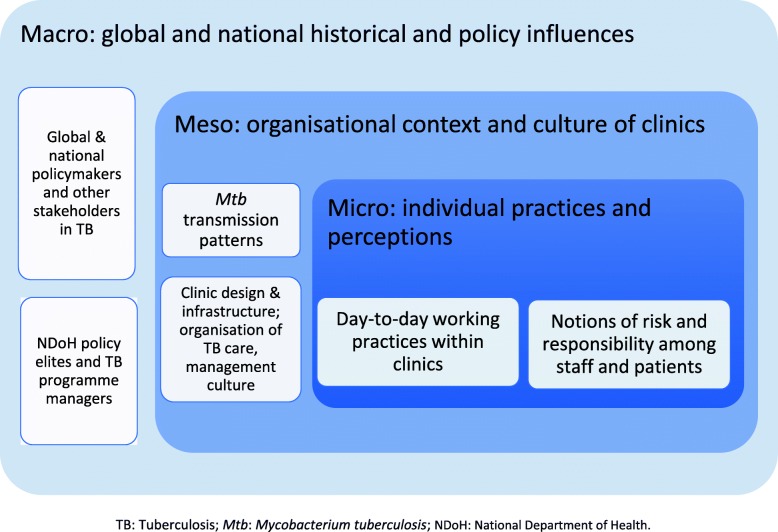
A whole systems approach to tuberculosis infection prevention and control

In the *Umoya omuhle* (“good air”) project, we adopt an interdisciplinary approach that 1) contextualises work processes and practices related to TB IPC at the clinic level within the structure and functioning of the whole system, and 2) analyses interactions across components. A whole systems approach addresses the dynamic interactions across macro-, meso-, and micro-levels of the health system (see Fig. 1): the project methodology covers both the health system ‘hardware’ of TB IPC, i.e., infrastructure, space, resources, and operational guidelines, as well as the health system ‘software’, i.e., actors’ norms, values, and work processes that help understand how principles of TB IPC are translated into practice [15].

Methods drawn from epidemiology, anthropology, and health policy and systems research are enabling rich contextual analysis of how nosocomial *Mtb* transmission occurs, as well as opportunities to address the problem holistically. A community-based social contact survey will provide data on how much person contact time occurs in clinics versus other settings, and a clinic-based TB prevalence survey will provide data on increased rates of clinic visiting in people with undiagnosed TB. This data will be used to parameterise a mathematical model of *Mtb* transmission. Using this model, we will estimate how much DS- and DR-TB result from transmission in health facilities versus other sites in the community. At the same time, in-depth case studies of TB-IPC practices and processes in six health facilities are shedding light on systems components pertinent to IPC including infrastructure, space, management and organisation of care. Working through provincial and district gatekeepers, and with consent of health facility managers and staff, we spent a few days in each of these facilities conducting observations, informal conversations, formal interviews and group discussions as well as structured assessments of patient flow and ventilation. Data on clinic design and the flow of people and air through clinic spaces will be juxtaposed with health workers’ accounts of risk in the context of their work, to contrast perceived and actual ‘hot spots’ of transmission risk, thus elucidating the ‘know-do’ gaps that exist in implementation of TB-IPC practices. Further, examining IPC policy and guidelines and health workers’ and managers’ perceptions of how ‘gold standards’ translate at lower levels of the health system will elucidate reasons for observed discrepancies between policy and practice. We use a System Dynamics Modelling approach to integrate quantitative and qualitative data gained through visual maps of the dynamic relationships across contextual factors, actors, and processes influencing the implementation of TB-IPC measures. This granular analysis is helping us to identify possible leverage points within the system and inform the design of targeted, data-driven interventions to reduce nosocomial *Mtb* transmission [16].

In turn, proposed interventions will be simulated and costed to provide decision-makers with better estimates of the impact of innovative systems approaches to IPC on *Mtb* transmission, including estimates of cost-effectiveness. The approach has potential for improving current methods to assess TB IPC implementation at facility level, providing additional criteria against which ‘underperforming’ facilities can be evaluated. For example, hitherto neglected domains of organisational culture, leadership, and patient- and workflow and their interaction in the everyday life of clinics could be usefully integrated into more in-depth assessment of TB IPC.

## Conclusions

A whole systems approach draws attention to both the human and the organisational dimensions of health care delivery. Elucidating the system and how it ‘works’ will improve our understanding of health managers and health workers’ motivations, fears, hopes, and capacity to adapt recommended TB-IPC measures to the real-life parameters of the clinics and policy environments they work in. In South Africa, emerging solutions targeting systemic change for improved IPC will need to be embedded within broader national initiatives to ‘re-engineer’ primary health care and improve healthcare facility performance. At the same time, our approach to the ‘problem’ of compromised IPC and identification of new strategies in the South African context holds promise for other initiatives intended to prevent nosocomial transmission of drug-resistant and other infections in a holistic and sustainable manner.

## Data Availability

Not applicable.

## Abbreviations

AMR: Antimicrobial resistance
DR-TB: Drug-resistant tuberculosis
DS-TB: Drug-sensitive tuberculosis
HCW: Health care worker
IPC: Infection prevention and control
*Mtb*: *Mycobacterium tuberculosis*
XDR: Extensively drug-resistant
